# Role of donor genotype in RT-QuIC seeding activity of chronic wasting disease prions using human and bank vole substrates

**DOI:** 10.1371/journal.pone.0227487

**Published:** 2020-01-07

**Authors:** Soyoun Hwang, Justin J. Greenlee, Eric M. Nicholson

**Affiliations:** United States Department of Agriculture, Agricultural Research Service, National Animal Disease Center, Virus and Prion Research Unit, Ames, Iowa, United States of America; Creighton University, UNITED STATES

## Abstract

Chronic wasting disease is a transmissible spongiform encephalopathy of cervids. This fatal neurodegenerative disease is caused by misfolding of the cellular prion protein (PrP^C^) to pathogenic conformers (PrP^Sc^), and the pathogenic forms accumulate in the brain and other tissues. Real-time Quaking Induced Conversion (RT-QuIC) can be used for the detection of prions and for prion strain discrimination in a variety of biological tissues from humans and animals. In this study, we evaluated how either PrP^Sc^ from cervids of different genotypes or PrP^Sc^ from different sources of CWD influence the fibril formation of recombinant bank vole (BV) or human prion proteins using RT-QuIC. We found that reaction mixtures seeded with PrP^Sc^ from different genotypes of white-tailed deer or reindeer brains have similar conversion efficiency with both substrates. Also, we observed similar results when assays were seeded with different sources of CWD. Thus, we conclude that the genotypes of all sources of CWD used in this study do not influence the level of conversion of PrP^C^ to PrP^Sc^.

## Introduction

Chronic wasting disease (CWD) is a form of transmissible spongiform encephalopathy (TSE) or prion disease. Prion diseases are a group of fatal neurologic diseases that result from the misfolding of the cellular prion protein (PrP^C^) into a pathogenic form (PrP^Sc^) in the brain. Prion diseases include CWD in cervids like deer, elk, moose, and reindeer; scrapie in sheep; bovine spongiform encephalopathy (BSE) in cattle; and Creutzfeldt-Jakob disease (CJD), fatal familial insomnia (FFI), Gerstmann-Sträussler-Scheinker syndrome (GSS), and kuru in humans. Misfolded proteins accumulate in the central nervous system in all TSEs, but in CWD cases, the misfolded prion proteins are distributed widely not only in the nervous system but also in lymphoid tissues, muscle, and blood [1–3]. Since prions are shed via saliva, urine, and feces from infected cervids, CWD can spread rapidly, and it also can affect free-ranging wild animals. In the United States, CWD has been reported in 24 states and it also has been reported in other countries including Canada, South Korea, Norway, Finland and most recently, Sweden [4–7].

The association of prion protein gene with TSEs is well documented with extensive research showing that the expression of a prion protein is necessary for a host to develop disease and that differences in the prion gene sequence altering disease susceptibility and incubation time [8–12]. In elk, codon 132 is polymorphic and can code for either methionine (M) or leucine (L), which can affect CWD incubation periods. For example, LL132 elk challenged with the CWD agent have incubation periods approximately 1.5 times longer than ML132 elk, and 3 times longer than MM132 elk [13]. In white-tailed deer (WTD), polymorphisms at codons 95 and 96 influence CWD susceptibility. Researchers found white-tailed deer with Q95H and G96S are under represented in the CWD positive populations [14].

We employed real-time quaking-induced conversion (RT-QuIC) to assess prion seeding activity using recombinant human or bank vole substrates. RT-QuIC is an efficient and sensitive tool to detect prions in various samples from humans and animals. Also, RT-QuIC has shown potential analytical applications such as prion strain discrimination, drug screening, and screening for prion contamination [15].

Limited reports utilizing *in-vitro* assays to test the transmissibility of CWD to human are available and no report has been published for investigating the effect of different genotypes of CWD with human substrate. In this study, we tested if different genotypes of CWD or different sources of CWD affect the seeding activity of BV and human rPrP substrates. We hypothesized that the CWD agent derived from hosts with different *PRNP* genotypes that are associated with short survival time in animal experiments may affect the seeding activity in RT-QuIC reactions.

## Materials and methods

### Ethics statement

The animal experiments from which the archived tissue samples used as RT-QuIC seed in this study were reviewed and approved by the National Animal Disease Center’s Institutional Animal Care and Use Committee (protocol numbers: 3451 and 3669). The animal experiments were carried out in accordance with the Guide for the Care and Use of Laboratory Animals (Institute of Laboratory Animal Resources, National Academy of Sciences, Washington, DC). The details of this study are described in citation numbers 16 and 17.

### Sources of inocula and RT-QuIC seed

Archived brain samples from CWD-infected white-tailed deer and reindeer were obtained from studies [16, 17] previously conducted at the National Animal Disease Center. Animals, genotypes, inoculum source and time to disease on set are summarized in Table 1 and Table 2.

**Table 1:** Animal experimental summary of genotype, inoculum, survival period, and EIA in white-tailed deer inoculated intracranially with the agent of chronic wasting disease from elk, white-tailed deer and mule deer.

Animal Number	Group Number	Eartag	Genotype		CWD Inoculum	Incubation period (mo.)	EIA O.D.
			95	96			
1	A	648	QQ	GG	WTD	17	3.27
2		628	QQ	GS	WTD	20.93	4.00
3		654	QQ	GS	WTD	23.47	4.00
4	B	676	QQ	GG	Elk	10.37	3.77
5		635	QQ	GG	Elk	17.17	3.97
6		646	QQ	GG	Elk	17.73	4.00
7		677	QQ	GS	Elk	24.1	4.00
8	C	680	QQ	GG	MD	14.5	4.00
9		639	QQ	GG	MD	16.1	4.00
10		643	QQ	GS	MD	22.13	4.00
11		682	QQ	GS	MD	26	4.00
12		681	QH	GS	Neg. control	20.17	0.07

**Table 2:** Animal experimental summary of genotype, inoculum, survival period, and EIA in reindeer inoculated intracranially with the agent of chronic wasting disease from elk, white-tailed deer and mule deer.

Animal Number	Group Number	Eartag	Genotype					CWD Inoculum	Incubation period (mo.)	EIA O.D.
			002	129	138	169	176			
1	A	568	VV	GG	NN	VV	NN	WTD	20.9	2.10
2		521	VV	GG	NS	VV	ND	WTD	33.9	3.76
3		567	VV	GG	NS	VV	NN	WTD	34.1	4.00
4		555	MV	SG	SS	MV	ND	WTD	53.3	3.03
5	B	514	VV	GG	NS	VV	NN	Elk	38.7	4.00
6		565	VV	GG	NS	VV	ND	Elk	41.7	1.11
7		571	na	SG	SS	MV	NN	Elk	42.2	1.32
8	C	502	VV	GG	NN	VV	NN	MD	24.8	4.00
9		524	VV	GG	SS	VV	DD	MD	31.0	2.79
10		552	VV	GG	NS	VV	ND	MD	43.5	1.16
11		530	VV	GG	NS	VV	NN	Neg. Control	34.1	0.07

### Enzyme Immunoassay (EIA) of brain homogenates from cervids

The IDEXX HerdChek EIA test kit was used to selectively detect the presence of disease associated misfolded prion protein. The IDEXX HerdCheck Assay can be used to test various mammalian tissues [18]. Any disease-associated conformer, PrP^Sc^, can bind to the ligand on the surface which is immobilized on the surface and captured with an antigen. Brain homogenates from CWD-infected cervid animals were assessed using the IDEXX HerdChek EIA kit in the absence of proteinase K digestion. EIA was performed as described by the manufacturer. The cutoff value was determined by the negative control sample provided by the manufacturer and the optical density value was around 0.07 ± 0.005. If the optical density value was over 0.15, the samples were considered positive. All brain samples were normalized with EIA kit before analysis by RT-QuIC by diluting to an O.D. around 1.0.

### Recombinant prion protein production and purification

*E*. *coli* (BL21(λDE3)) was transformed with the pET28a vector containing the BV PrP gene (amino acids 23–231; GenBank accession number AF367624) and human PrP (amino acids 90–231, with M at residue 129; GenBank accession number CAA58442.1). The recombinant prion proteins were expressed and purified as described by Vrentas *et al* [19]. The concentration of pooled protein eluent was measured by UV and calculated from the absorbance at 280 nm using an extinction coefficient of 62005 or 20800 M^-1^cm^-1^ as calculated for BV (23–231) and human (90–231) rPrP.

### RT-QuIC protocol

RT-QuIC reactions were performed as previously described [20–26]. The reaction mix was composed of 10 mM phosphate buffer (pH 7.4), 100 mM NaCl (for human rPrP substrate) or 300 mM NaCl (BV rPrP substrate), 0.1 mg/ml recombinant BV or human prion proteins, 10 μM thioflavin T (ThT), and 1 mM ethylenediaminetetraacetic acid tetrasodium salt (EDTA). Aliquots of the reaction mix (98 μL) were loaded into each well of a black 96-well plate with a clear bottom (Nunc, Thermo Fisher Scientific) and seeded with 2 μL of brain homogenate dilutions. The plate was then sealed with plate sealer film and incubated at 42°C in a BMG FLUOstar Omega plate reader with cycles of 15 min shaking (700 rpm double orbital) and 15 min rest for 100 h. ThT fluorescence measurements (excitation, 460 nm; emission 480 nm, bottom read, 20 flashes per well, manual gain 1400) were taken every 45 min.

All reactions for each dilution and each sample were performed in 8 replicates of RT-QuIC assays. ThT fluorescence data are displayed as the average ThT fluorescence of four technical replicates for each time point and, to be considered positive, the ThT fluorescence of at least two replicate reactions must be positive. As previously described for classification of positive samples by RT-QuIC, the positive threshold was calculated as the mean value of non-inoculated control sheep brain homogenates plus 10 standard deviations [21, 27, 28].

## Results

### Quantitation of PrP^Sc^ by EIA in brain samples from negative control and CWD infected cervids

Brainstem samples (obex) were collected from cervids with clinical signs of CWD. All of these brain samples have been studied previously [16, 17]. EIA was performed on these brainstem samples of white-tailed deer (Table 1) and reindeer (Table 2) to determine the relative amount of misfolded prion protein in the sample.

### RT-QuIC reactions seeded with brain material from CWD infected white-tailed deer (WTD) using recombinant BV PrP substrate

**Fig 1:**
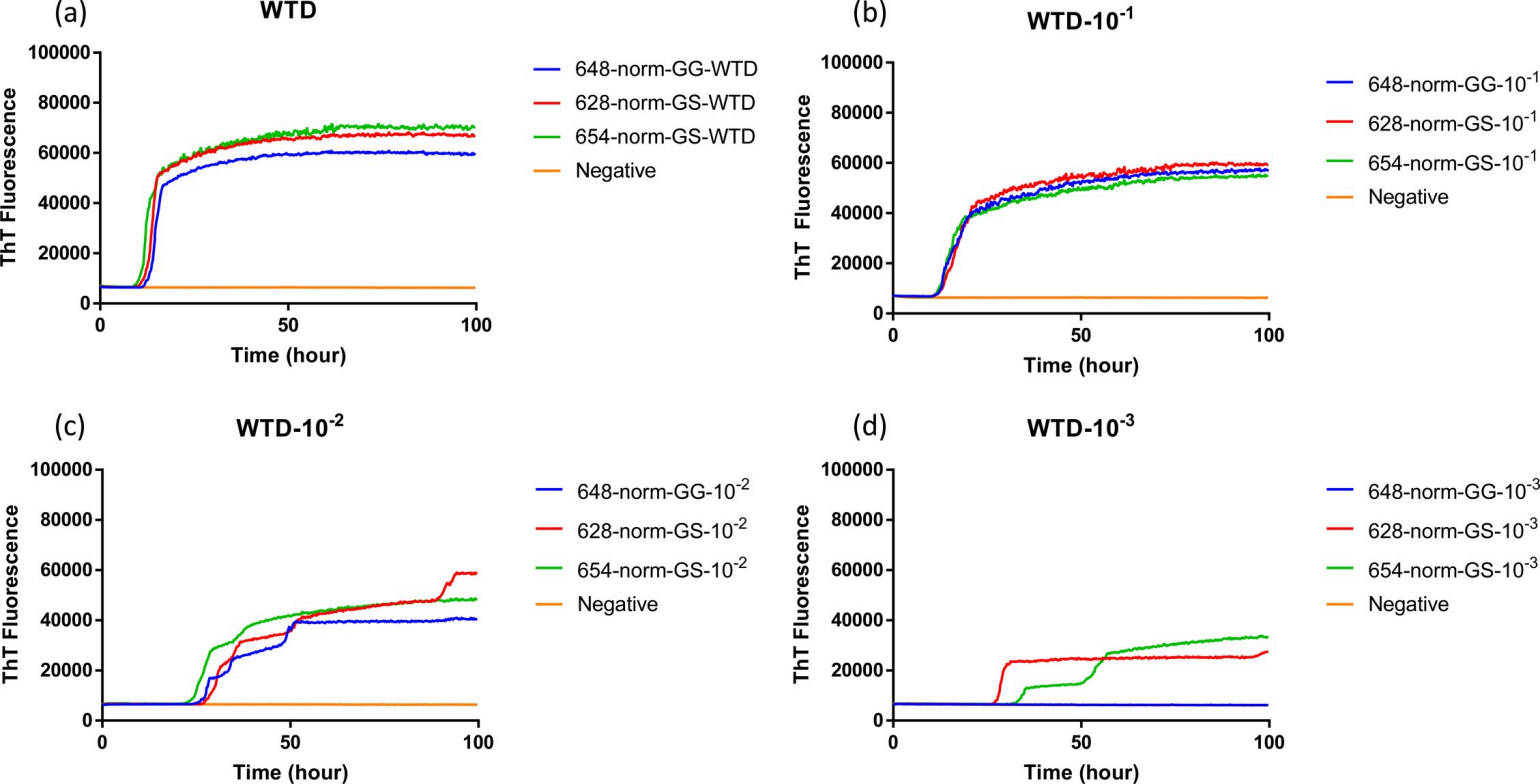
RT-QuIC reactions seeded with CWD infected or not infected white tailed deer brains using BV rPrP as a substrate. RT-QuIC reactions were seeded with EIA normalized stock (a), 10^−1^ (b), 10^−2^ (c), and 10^−3^ (d) dilutions of three CWD infected WTD brains and one negative animal. All reactions were seeded with brain homogenates of WTD with the addition of 0.001% of SDS. Shown are the average ThT fluorescence readings (thick lines) with standard deviations (thin lines) determined from all replicates (four replicate reactions per each animal).

**Fig 2:**
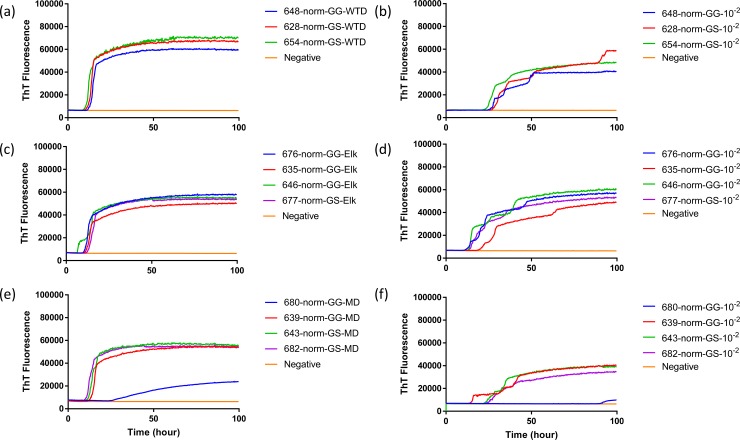
**Comparison of seeding activity of RT-QuIC reactions with BV rPrP which contain seeds from WTD inoculated with brains of infected WTD (a and b), elk (c and d) or mule deer (e and f).** Left panel shows RT-QuIC reactions seeded with EIA normalized brains and right panel shows RT-QuIC reactions seeded with 10^−2^ dilutions of brains. Data are presented as mean ThT fluorescence of 4 repeated reactions.

**Fig 3:**
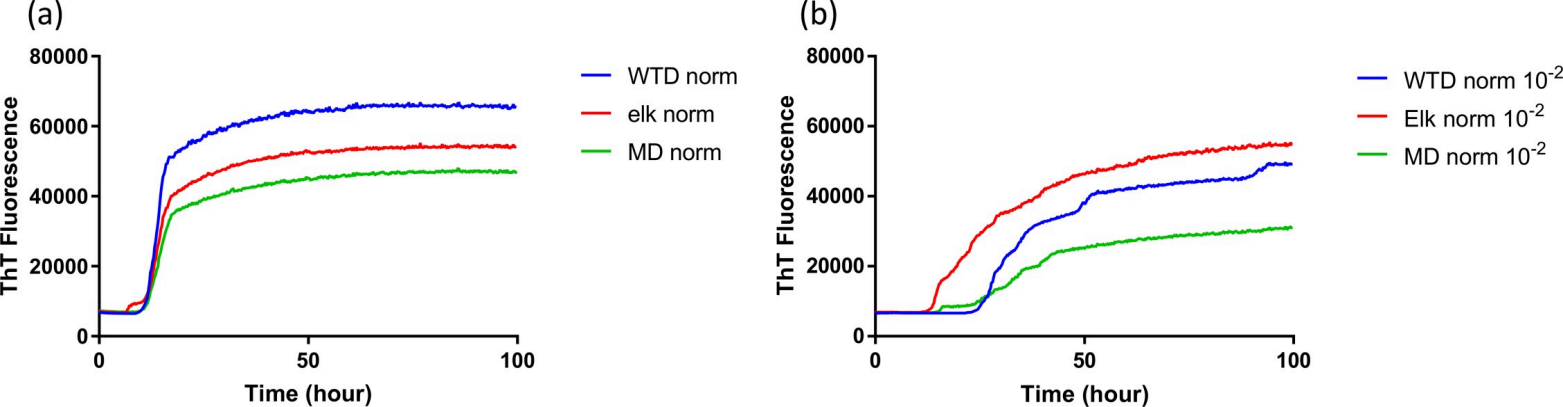
Comparison of averaged seeding activity of RT-QuIC reactions with BV rPrP seeded with brains from WTD inoculated with brains from different cervid species. (WTD: blue line, elk: red line, MD: green line). All assays seeded with (a) EIA normalized stock and (b) 10^−2^ dilution of WTD brains inoculated with each CWD source was averaged and compared to assays seeded with two other CWD inocula. Data are presented as mean ThT fluorescence of 4 repeated reactions.

To evaluate the influence that *PRNP* genotype of the white-tailed deer and source of CWD inoculum on the seeded conversion of bank vole (BV) rPrP, RT-QuIC reactions were seeded with different dilutions (10^−1^ to 10^−3^) of EIA normalized brain tissues. Assays seeded with CWD^WTD^, CWD^elk^, and CWD^MD^ showed increase of ThT fluorescence of positive white-tailed deer but no ThT increase for a negative control. First, brain dilutions were tested in RT-QuIC assays for an optimal dilution for comparison between seeds from different genotypes. From EIA normalized stock to 10^−2^ brain dilutions seemed optimal to compare each other. Assays seeded with normalized brain seed without dilution showed a short lag time and this lag time increased as the brain samples were further diluted (Fig 1). RT-QuIC reactions seeded with CWD^WTD^, CWD^elk^ and CWD^MD^ were compared based on the *PRNP* genotype at codon 96, GG96 and GS96, influence on seeding activity, but no relevant difference was found (Fig 2). Also, all assays from each CWD source were averaged and compared to other assays seeded with another CWD inoculum (Fig 3). There was no substantial difference in lag time in the assays seeded with normalized brain, but assays seeded with 10^−2^ brain dilution showed that average lag time of elk was shorter than the lag time seeded with two other species. It is worth noting that assays seeded with CWD^MD^ have overall lower ThT fluorescence, a difference that could be indicative of reduced fibril formation (Fig 3).

### RT-QuIC reactions seeded with brain material from from CWD infected white-tailed deer (WTD) using recombinant human PrP substrate

**Fig 4:**
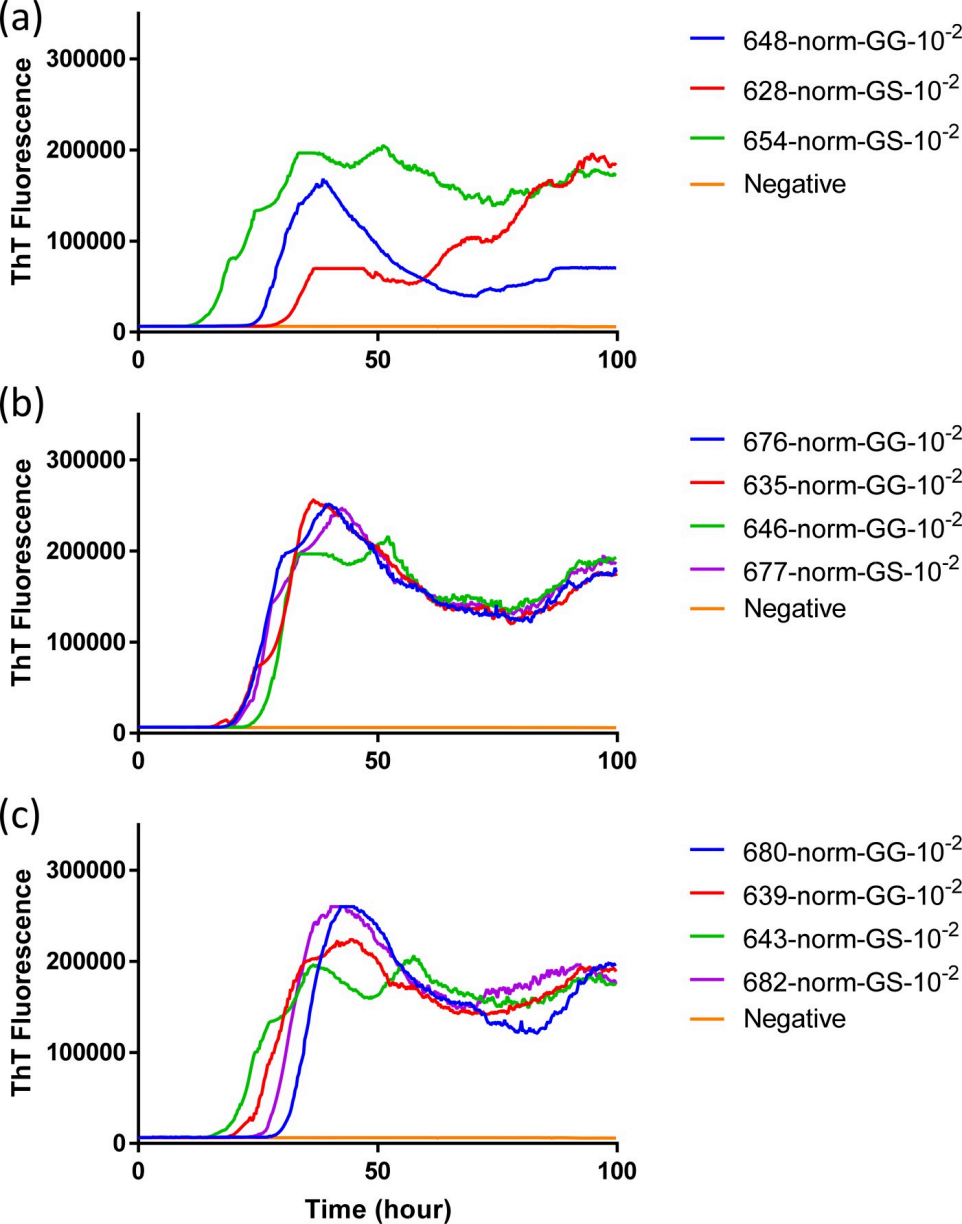
**Comparison of seeding activity of RT-QuIC reactions with human rPrP which contain brain seeds from WTD inoculated with infected brains from WTD (a), elk (b) or mule deer (c).** This shows RT-QuIC reactions seeded with 10^−2^ dilution of EIA normalized brains in the presence of 0.002% SDS. Data are presented as mean ThT fluorescence of 4 repeated reactions. Data are presented as mean ThT fluorescence of 4 repeated reactions.

**Fig 5:**
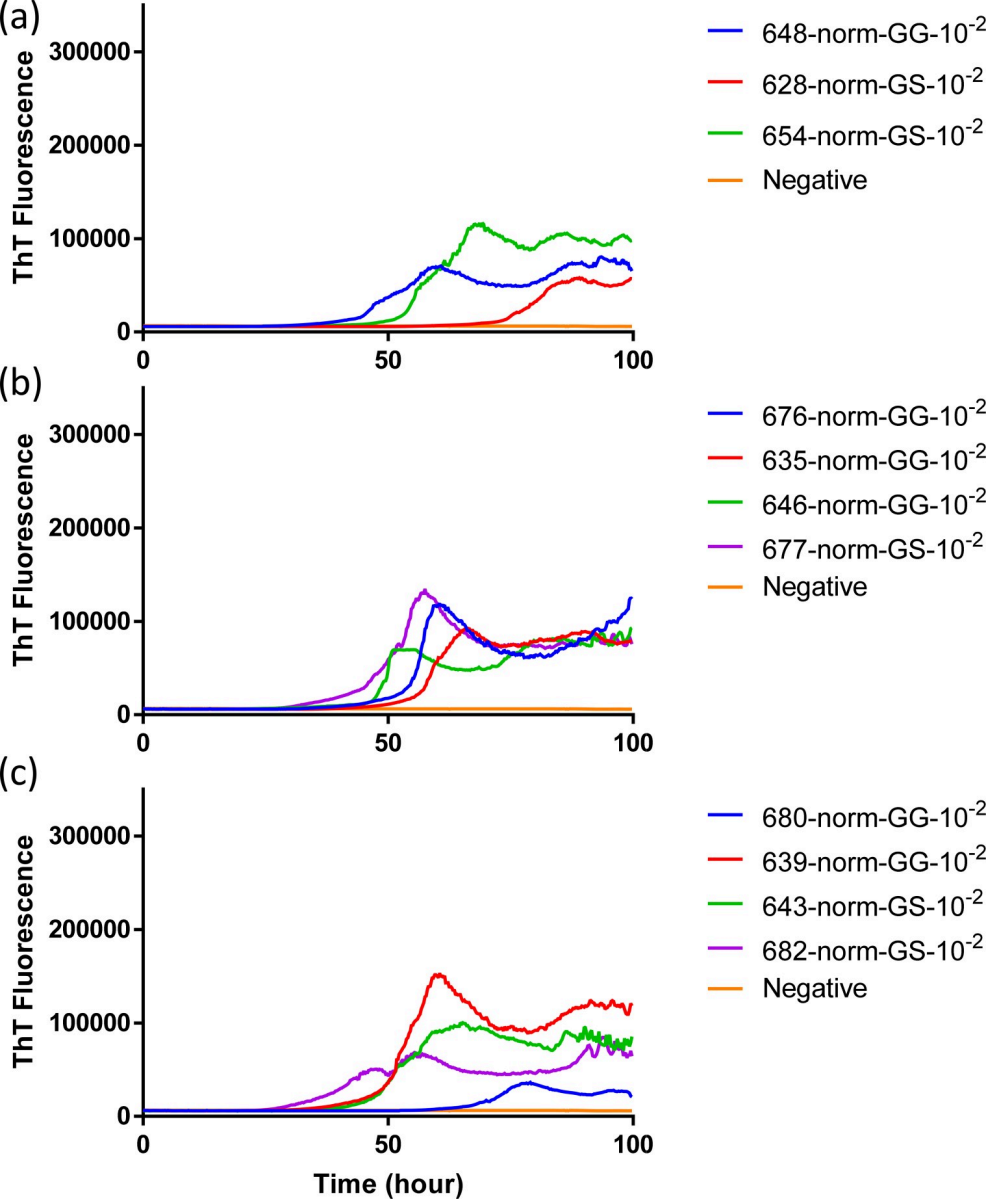
**Comparison of seeding activity of RT-QuIC reactions with human rPrP which contain brain seeds from WTD inoculated with brains of infected white-tailed deer (a), elk (b) or mule deer (c) in the presence of 0.001% SDS.** This shows RT-QuIC reactions seeded with 10^−2^ dilution of EIA normalized brains. Data are presented as mean ThT fluorescence of 4 repeated reactions. Data are presented as mean ThT fluorescence of 4 repeated reactions.

**Fig 6:**
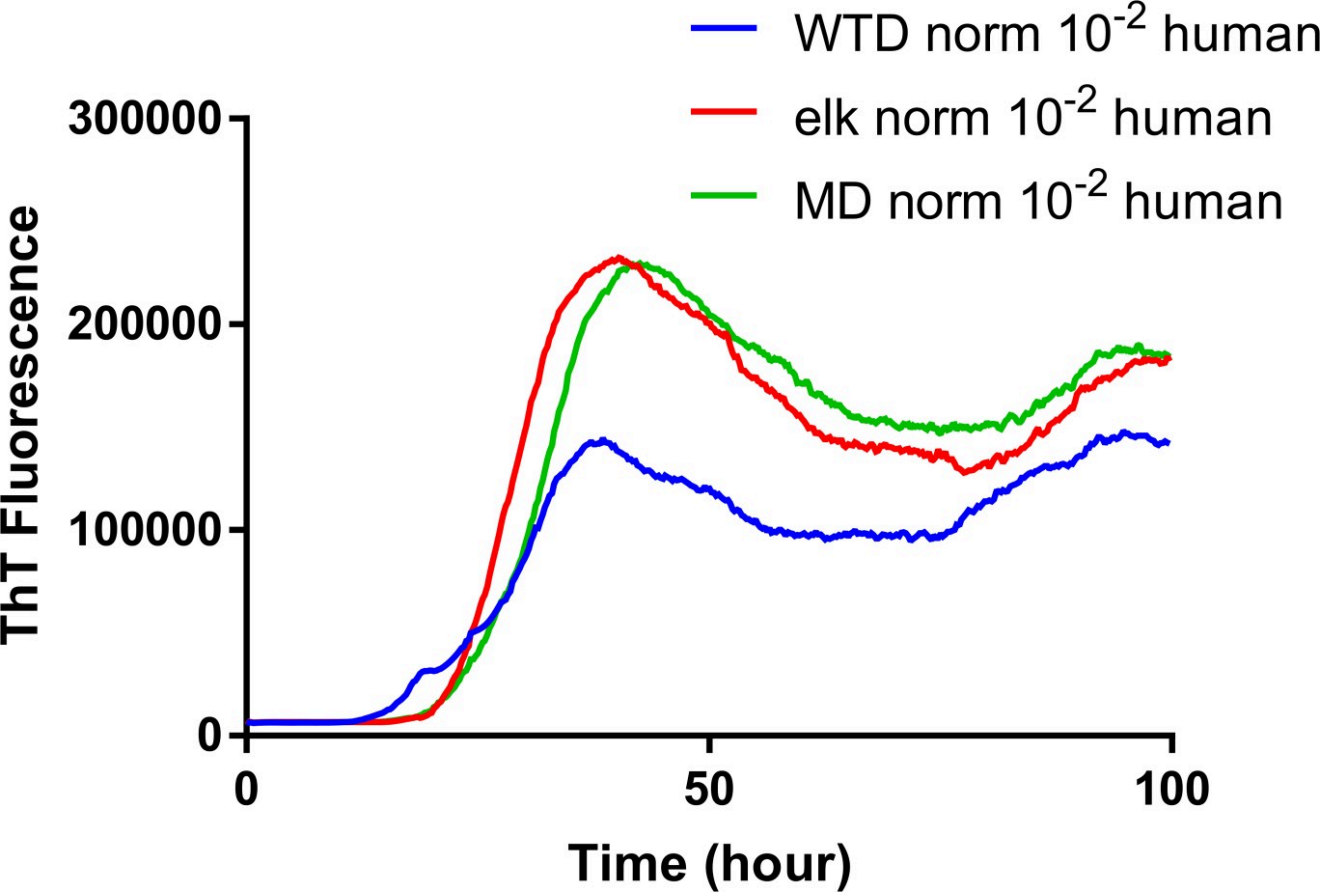
Comparison of averaged seeding activity of RT-QuIC reactions with human rPrP seeded with brains from WTD inoculated with brains of infected white-tailed deer, elk, or mule deer. (WTD: blue line, elk: red line, MD: green line). All assays from each CWD source was averaged and compared to assays seeded with different CWD inocula. Data are presented as mean ThT fluorescence of 4 repeated reactions.

To evaluate the influence that *PRNP* genotype of the white-tailed deer and source of CWD inoculum on the seeded conversion of recombinant human PrP, RT-QuIC reactions were seeded with 10^−2^ of EIA normalized brain tissues based on the data from optimal dilution tests with bank vole (BV) rPrP. All assays containing positive seed showed ThT fluorescence increase within 30 hours indicating that recombinant human PrP can be useful to detect CWD prions as can be seen in Fig 4. Assays seeded with CWD^WTD^ showed different seeding activity between animals but there was no consistent association between seeding activity and genotypes of animals used as the seed. Assays seeded with CWD^Elk^ and CWD^MD^ similarly did not exhibit relevant differentiation between different genotypes of animals, GS96 vs GG96. Different SDS concentration were assessed with regard to differentiation of seeding activity of these animals, but overall ThT intensity was reduced and lag time was increased suggesting that this reaction condition is not optimal for conversion or differentiation (Fig 5). All reaction assays from each CWD source were averaged across genotypes and compared to other assays seeded with other CWD inocula in white-tailed deer as displayed in Fig 6. Assays seeded with CWD^MD^ and CWD^Elk^ showed similar seeding activity in terms of ThT fluorescence and lag time, but assays seeded with CWD^WTD^ showed lower ThT fluorescence although they exhibited a similar lag time to that observed for the other two sources of CWD.

### RT-QuIC reactions seeded with brain material from CWD infected reindeer using recombinant BV PrP substrate

**Fig 7:**
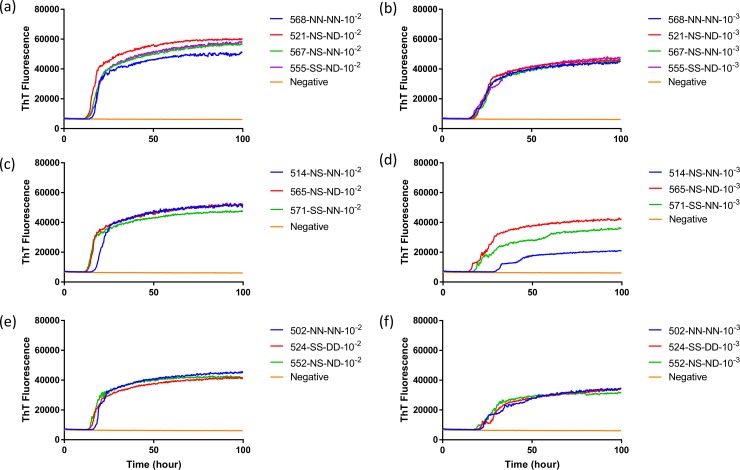
**Comparison of seeding activity of RT-QuIC reactions with BV rPrP which contain seeds from reindeer inoculated with brains of infected WTD (a and b), elk (c and d) or mule deer (e and f).** Left panel shows RT-QuIC reactions seeded with 10^−2^ brain dilution of EIA normalized and right panel shows RT-QuIC reactions seeded with 10^−3^ dilutions of brains. Data are presented as mean ThT fluorescence of 4 repeated reactions.

**Fig 8:**
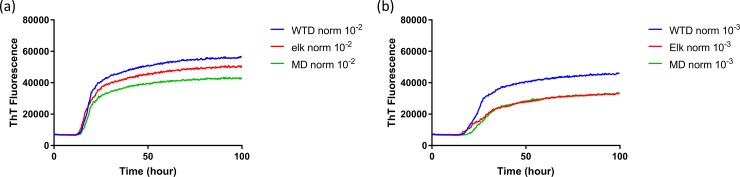
Comparison of averaged seeding activity of RT-QuIC reactions with BV rPrP seeded with brains from reindeer inoculated with brains of infectious cervid species. (WTD: blue line, elk: red line, MD: green line). All assays seeded with (a) 10^−2^ or (b) 10^−3^ dilution of EIA normalized stock dilutions of reindeer brains inoculated with each CWD source was averaged and compared to assays seeded with two other CWD inocula. Data are presented as mean ThT fluorescence of 4 repeated reactions.

To evaluate the influence that *PRNP* genotype of the reindeer and source of CWD inoculum had on the seeded conversion of recombinant BV PrP, RT-QuIC reactions were seeded with different dilutions (10^−2^ to 10^−3^) of EIA normalized brain tissues. Assays seeded with CWD^WTD^, CWD^elk^, and CWD^MD^ showed an increase in ThT fluorescence of positive reindeer within 20 hours for most samples but no ThT increase for a negative control. RT-QuIC reactions seeded with CWD^WTD^, CWD^elk^ and CWD^MD^ were compared to determine if *PRNP* genotypes at residue 138 influence the seeding activity, but no discernible difference was identified (Fig 7). Previously reported study showed that reindeer with the NN138 polymorphism had the shortest survival times in intracranially inoculated groups [17]. Also, all assays from each CWD source were averaged across genotypes and compared to other assays seeded with other CWD inocula in reindeer (Fig 8). Given the published differentiation of TSE isolates based upon fluorescence intensity by RT-QuIC it is worth noting that at a 10^−2^ dilution all three sources CWD exhibited similar seeding activity based on both ThT fluorescence intensity and lag time, but with further dilution (10^−3^) CWD^WTD^ exhibited a slightly higher ThT fluorescence than assays seeded with the other two sources of CWD. Given the similarities at the lower dilution we do not believe this to be a meaningful observation but simply note it here for completeness.

### RT-QuIC reactions seeded with brain material from CWD infected reindeer using recombinant human PrP substrate

**Fig 9:**
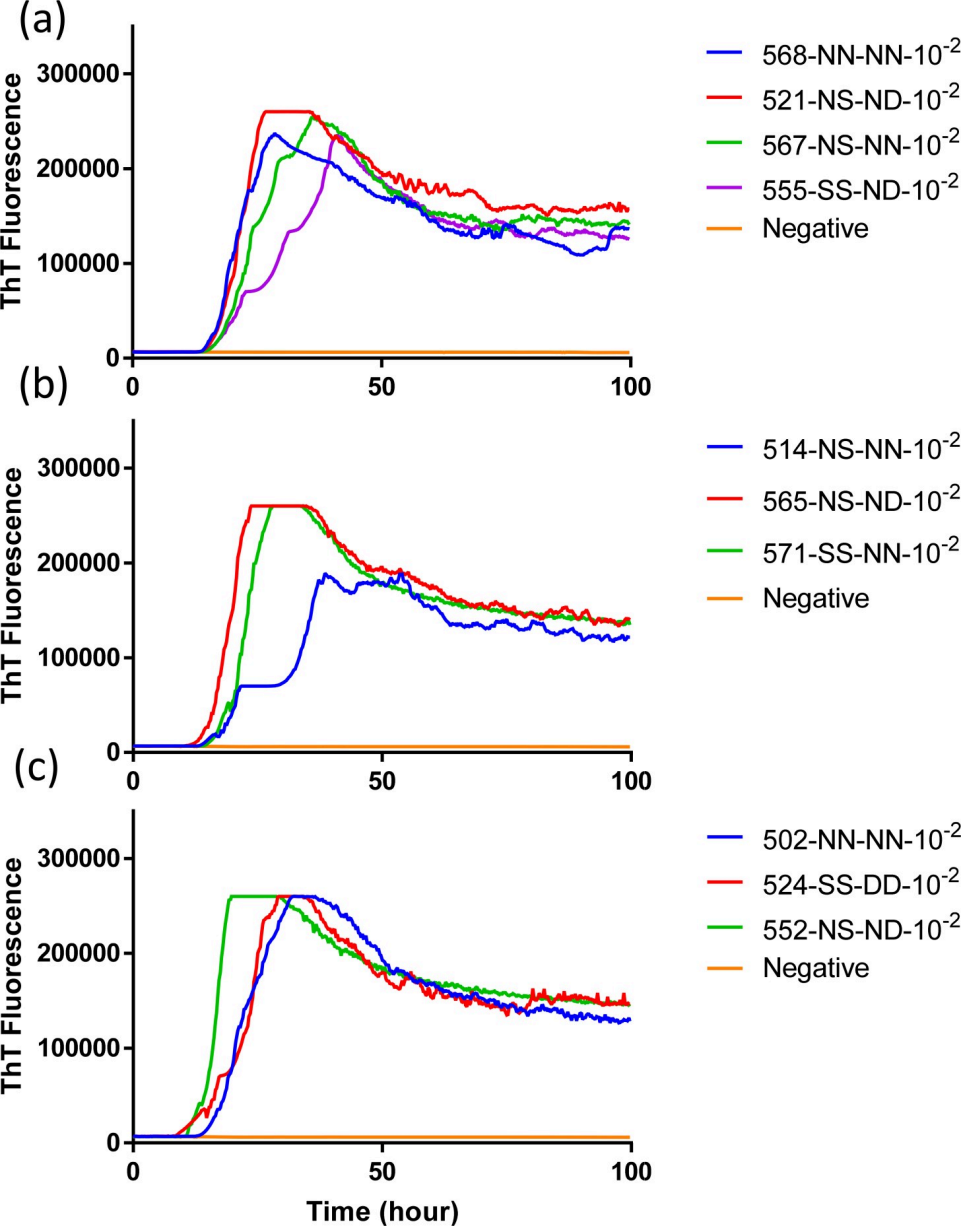
**Comparison of seeding activity of RT-QuIC reactions with human rPrP which contain brain seeds from reindeer inoculated with brains of infectious WTD (a), elk (b) or mule deer (c).** This shows RT-QuIC reactions seeded with 10^−2^ dilution of EIA normalized brains in the presence of 0.002% SDS. Data are presented as mean ThT fluorescence of 4 repeated reactions. Data are presented as mean ThT fluorescence of 4 repeated reactions.

**Fig 10:**
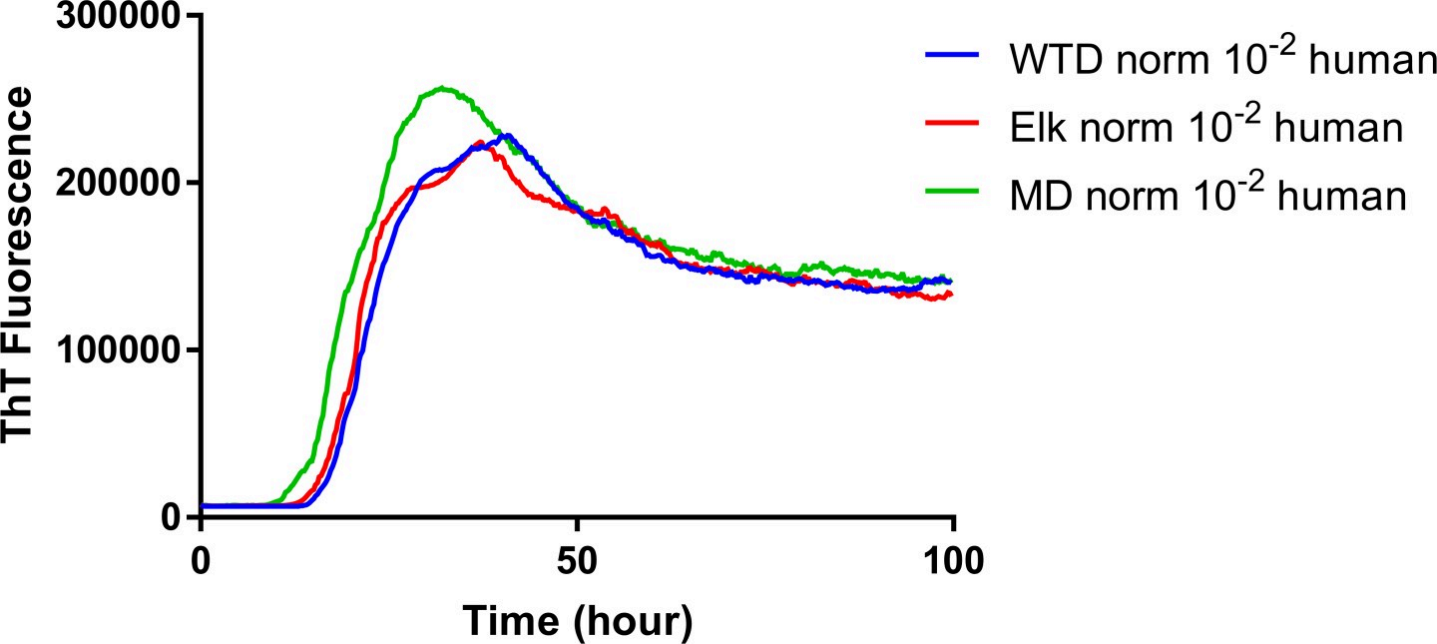
Comparison of averaged seeding activity of RT-QuIC reactions with human rPrP seeded with 10^−2^ dilution of EIA normalized brains from reindeer inoculated with brains of infectious cervid species. (WTD: blue line, elk: red line, MD: green line). All assays from each CWD source was averaged and compared to assays seeded with different CWD inocula. Data are presented as mean ThT fluorescence of 4 repeated reactions.

In order to evaluate the influence that *PRNP* genotype of the reindeer and source of CWD inoculum had on the seeded conversion of recombinant human PrP, RT-QuIC reactions were seeded with 10^−2^ of EIA normalized brain tissues from CWD infected reindeer. All assays seeded with positive animals showed ThT fluorescence increase within 20 hours similar to the results reported here for assays seeded with CWD positive white-tailed deer confirming that recombinant human PrP can be useful for rapid detection of CWD prions (Fig 9). In addition, all reaction assays from each CWD source were averaged across genotypes and the result of which are shown in the figure (Fig 10). All reactions had similar lag time and ThT fluorescence intensity as observed with seeded reactions from white-tailed deer which suggests that there is no consistent association between *in-vitro* seeding activity and genotypes of reindeers or source of CWD inoculum.

## Discussion and conclusions

Using *in-vitro* RT-QuIC assays, we have previously shown the impact of scrapie seed derived from different genotypes of sheep on RT-QuIC seeding activity [29]. In the present study, we have further identified the effect of different genotypes and species of cervids previously inoculated with different sources of CWD in the seeding activity in RT-QuIC using BV and human recombinant prion protein as the substrate. Previously we have shown that RT-QuIC reactions seeded with VRQ/VRQ sheep scrapie had higher seeding activity with shorter lag time compared to assays seeded with ARQ/ARQ sheep scrapie. In addition to RT-QuIC assays, bioassay confirmed that mice inoculated with VRQ/VRQ sheep scrapie had a shorter incubation period [29]. Since this previous study confirmed that *in-vitro* seeding activity can accurately reflect *in-vivo* results with regard to disease, we wanted to test how different genotypes of cervids or different source of CWD affect the seeding activity of recombinant BV PrP and human PrP. In this way, we may gain understanding of genotype effects on CWD transmission to human.

Using samples from previous studies, summarized in Tables 1 and 2, we assessed the seeded conversion of human rPrP in addition to BV rPrP by seeding with CWD prions from different sources. Based on the reputation of BV rPrP as a universal substrate in RT-QuIC [20, 30, 31], it was expected that we would observe efficient conversion of the samples tested here, of note is the observation that the efficiency of conversion was essentially indistinguishable regardless of CWD isolate or genotype among those tested further supporting the use of BV rPrP as a substrate in the detection of CWD.

Utilizing the same set of samples we also demonstrated that CWD prions sourced from WTD, elk or mule deer can seed human rPrP with high efficiency. To date, we are aware of only one report on the seeding activity of human substrate with CWD prions using RT-QuIC. This report showed that CWD prions from white-tailed deer could be used to seed human substrate (129M) and that the CWD conversion was more efficient than conversion with classical BSE prions [30]. Our study reconfirms that CWD is an efficient seed for human rPrP substrate. The relatively high efficiency of RT-QuIC amplification of CWD prions using human rPrP substrate may seem counter to the status of the broader understanding of CWD not being associated with human disease. This understanding is based on the published body of work on CWD and human transmission from either PMCA based amplification, transgenic mouse studies and non-human primates [31–37]. Collectively this work indicates that the species barrier is strong between humans and cervid CWD, and the efficient converstion of CWD prions using human rPrP by RT-QuIC does not challenge this because RT-QuIC exclusively assess the primary structure compatibility of the substrate with that of the secondary and tertiary structure of the seed. However, RT-QuIC does offer us a means to rapidly assess large numbers of amino acid substitutions between and within species associated with CWD to determine whether. In this study we did not observe changes in the key parameters to differentiate RT-QuIC (lag time, amyloid formation, or ThT fluorescence signal intensity) [38] based on the genotypes of CWD or with different sources of CWD. Since the RT-QuIC assay monitors the fibril formation in real-time via binding of the fluorescence marker ThT to the amyloid fibrils, different lag times indicate different seeding rate of reactions, which is often used to differentiate the reaction. The maximal ThT fluorescence intensity of each reaction can be a simple indicator to reflect seeding activity measured in the RT-QuIC [39]. However, in this study, the source genotype of the CWD seed from either white-tailed deer or reindeer did not seem to show any distinct correlation with reaction time in RT-QuIC when analyzed with lag time and ThT fluorescence intensity. One possible explanation for these results might be that the original inoculum, used to i.c. inoculate the WTD and reindeer used as seeds for these experiments, was pooled brain material (CWD affected elk (CWD^elk^), CWD-affected mule deer from Wyoming (CWD^md^), or CWD from white-tailed deer from Wisconsin combined with brain material from experimentally challenged white-tailed deer (CWD^wtd^)), therefore it might contain a mixture of CWD strains and thus not exhibit a meaningful difference by RT-QuIC.

Haley and colleagues provide a comprehensive evaluation of RT-QuIC conversion of different genotypes of cervid CWD seed using various genotypes of cervid rPrP advance the understanding of genotype based susceptibility/resitance of CWD [40], and Orru and colleagues conducted an extensive assessment of RT-QuIC derived fibrils to further the understanding of strains using RT-QuIC and detailed biochemical characterization of the resultant fibril products [20]. The existence of strains and CWD source genotype are components in this study, however, the focus of the work is whether the BV prion protein is equivalently sensitive at the detection of CWD from white-tailed deer and reindeer of different genotypes and whether these different genotypes might be of different risk to humans through differences in the seeding capacity. In this report we showed that the recombinant BV prion protein substrate exhibits equivalent sensitivity for the different species and genotypes studied here further supporting the use of BV prion protein as a universal substrate in the development of RT-QuIC based diagnostics, and the rate of conversion of PrP^C^ to PrP^Sc^ using human substrate does not differ based on the species or genotype of those tested here. This *in vitro* assay only assesses the seeded misfolding aspect of the complex disease process of TSEs, and it does not specifically report on the disease susceptibility/resistance of humans to CWD. For this data on evaluating seeded conversion, there is no evidence supporting greater or lesser risk with regard to human transmission from CWD of different species or genotypes.
